# Supplementary material for: Effectiveness of different recruitment strategies in an RCT of a surgical device: experience from the Endobarrier trial
Source: BMJ Open. 2019 Nov 14;9(11):e032439. doi: 10.1136/bmjopen-2019-032439 (PMC6886935; doi:10.1136/bmjopen-2019-032439)
Supplement: Supplementary data [file bmjopen-2019-032439supp001.pdf]

EndoBarrier Telephone Screening Form

Date 24.04.2014
Version 1.0

Date: |__|__| / |__|__| / |__|__| (DD/MM/YY)

Introduce yourself and explain purpose of the call. Explain that they will be asked details of their medications so please collect them now or organise another time to call if unavailable.

Personal details

Name: ……………………………………..

Telephone numbers: **Home** …………………………………
Work …………………………………
Mobile …………………………………

E-mail ………………………………………………………………………

Date of birth: ___ /___ /______

How did you hear about the study?

GP letter	☐	which surgery?………………………………..
GP surgery leaflet	☐	which surgery?……………………………….
Routine Care Provider	☐	who?……………………………….
Poster	☐	where?……………………………….
Newspaper advert	☐	where?……………………………….
Study website	☐	where?……………………………….
Diabetes Research Network	☐	who?……………………………….
Twitter/facebook	☐	who?……………………………….
Word of mouth	☐	who? (optional)……………………..
Other	☐	how?………………………………..

Short explanation of study…..

It is important for you to know that if you do enter the study you are free to withdraw at any time.

Would you like me to explain the study in greater detail for you?

Yes ☐ No ☐

Does the EndoBarrier study sound like something you think that you would be able to commit to?

Yes ☐ No ☐

If no what reason is given?………………………………………………………………
…………………………………………………………………………………………………
…………………………………………………………………………………………………

Screening questions

1. *Are you aged 18-65 years?* Yes ☐ No ☐⇒ ***STOP***

2. *Do you have type 2 diabetes mellitus?* Yes ☐ No ☐⇒ ***STOP***
   Have you had diabetes for at least 1 year? Yes ☐ No ☐⇒ ***STOP***
   Are you on any medication for your diabetes? Yes ☐ No ☐⇒ ***STOP***

   Do you use Insulin? Yes ☐⇒ ***STOP*** No ☐

3. *What is your weight?* ___________kg/lbs/stones & oz
   What is your height? ___________m/cm/feet & inches

Interviewer to calculate BMI: Is BMI: 30-50kg/m2 Yes ☐
No ☐
Requires further checks/confirmation ☐

Details……………………………………………………………………………………………..
…………………………………………………………………………………………………

4. *Do you have any medical conditions other than diabetes? (E.g. asthma, hypertension)*
Yes ☐ No ☐

If yes, what?…………………………………………………………………………………
…………………………………………………………………………………………………..
…………………………………………………………………………………………………

5. *Please tell us about all medications you have taken over the last 3 months (list all).*

Medications (names only, dosages not required)
…………………………………………………………………………………………..
…………………………………………………………………………………………
…………………………………………………………………………………………
…………………………………………………………………………………………
…………………………………………………………………………………………

6. *Have you ever had surgery on your stomach, intestines or colon/bowel before?*

Yes ☐
No ☐
Requires further checks/confirmation ☐

Details…………………………………………………………………………………..
…………………………………………………………………………………………..
…………………………………………………………………………………………

7. *Are you registered with a GP?* Yes ☐ No ☐⇒ ***STOP***

Explain that in order to proceed to the face-to-face screening visit, patient will be required to read the full participant information sheet (will be sent to them). Also, if they have not already done so, they must complete and return the consent form to the study team which allows contact with GP. This will allow us to gain essential information on their medications and health prior to the screening appointment.

Are you still interested in taking part in the EndoBarrier study?
Yes ☐ No ☐⇒ ***STOP***

If no what reason is given?………………………………………………………………
…………………………………………………………………………………………
…………………………………………………………………………………………….

If yes - you will need to attend a screening visit, where you will have blood taken. You need to be fasted for this visit. Please do not eat or drink anything on the morning of the visit and do not take any medications on that morning (but bring them with you).

8. *Do you consent to do this?* Yes ☐ No ☐⇒ ***STOP***

If volunteer has consented to face-to-face screening visit and is eligible:

- *Thank the caller for their time and arrange to send a full participant information sheet. They must read this before their screening appointment.*
- *Offer an appointment to attend the clinic to undergo screening and investigations, this will be fasted.*
- *Remind patient to return consent form to all us to contact GP if not already done so.*
- *Please bring along all medications to screening appointment.*

Outcome of pre-screening:

Ineligible to partake ☐ Reason ineligible....................................
Declined to take part ☐ Reason offered ....
If interested, has a summary patient information sheet(s) been sent?
No ☐
Yes ☐
If interested, has a patient information sheet(s) been sent?
No ☐
Yes ☐
Agreed plan for follow up..
....
